# Mitochondrial RNA Expression and Single Nucleotide Variants in Association with Clinical Parameters in Primary Breast Cancers

**DOI:** 10.3390/cancers10120500

**Published:** 2018-12-09

**Authors:** Marjolein J. A. Weerts, Marcel Smid, John A. Foekens, Stefan Sleijfer, John W. M. Martens

**Affiliations:** Department of Medical Oncology and Cancer Genomics Netherlands, Erasmus MC Cancer Institute, Erasmus University Medical Center, 3015 CE Rotterdam, The Netherlands; m.smid@erasmusmc.nl (M.S.); j.foekens@erasmusmc.nl (J.A.F.); s.sleijfer@erasmusmc.nl (S.S.); j.martens@erasmusmc.nl (J.W.M.M.)

**Keywords:** primary breast cancer, mitochondrial RNA variants, mitochondrial expression, clinicopathological markers

## Abstract

The human mitochondrial DNA (mtDNA) encodes 37 genes, including thirteen proteins essential for the respiratory chain, and RNAs functioning in the mitochondrial translation apparatus. The total number of mtDNA molecules per cell (mtDNA content) is variable between tissue types and also between tumors and their normal counterparts. For breast cancer, tumors tend to be depleted in their mtDNA content compared to adjacent normal mammary tissue. Various studies have shown that primary breast tumors harbor somatic mtDNA variants. A decrease in mtDNA content or the presence of somatic variants could indicate a reduced mitochondrial function within breast cancer. In this explorative study we aimed to further understand genomic changes and expression of the mitochondrial genome within breast cancer, by analyzing RNA sequencing data of primary breast tumor specimens of 344 cases. We demonstrate that somatic variants detected at the mtRNA level are representative for somatic variants in the mtDNA. Also, the number of somatic variants within the mitochondrial transcriptome is not associated with mutational processes impacting the nuclear genome, but is positively associated with age at diagnosis. Finally, we observe that mitochondrial expression is related to ER status. We conclude that there is a large heterogeneity in somatic mutations of the mitochondrial genome within primary breast tumors, and differences in mitochondrial expression among breast cancer subtypes. The exact impact on metabolic differences and clinical relevance deserves further study.

## 1. Introduction

Mitochondria are small organelles involved in multiple cellular processes. They are most renowned for their role in energy production, since they contain their own circular genomic entity encoding proteins essential for the respiratory chain and thereby for generating cellular ATP via oxidative phosphorylation. The human mitochondrial DNA (mtDNA) is gene-dense consisting of ~16569 base pairs encoding 37 genes: thirteen proteins, and two rRNAs and twenty-two tRNAs functioning in the mitochondrial translation apparatus. Polycistronic transcription of mtDNA is initiated at the non-coding D-loop region, and the resultant precursor transcripts are processed by excision of the tRNA genes (“tRNA punctuation model” [1]) generating individual mitochondrial tRNA, rRNA and mRNA transcripts. The total number of mtDNA molecules per cell (mtDNA content) is variable between tissue types, and interestingly also between tumors and their normal counterparts [2]. For breast cancer specifically, tumors tend to have reduced mtDNA content compared to adjacent normal mammary tissue [2,3,4,5,6,7,8,9,10], and mtDNA content in breast tumors positively correlates with the expression of mtDNA-encoded genes [11]. Decreased content and expression of mtDNA could indicate a reduced mitochondrial oxidative phosphorylation function within breast cancer, in line with the Warburg hypothesis [12] limiting energy production largely to glycolysis. Recently, we have shown mtDNA content to be associated with breast cancer patient outcome [13,14], underlining the clinical relevance of mitochondria in breast cancer.

Apart from mtDNA content, the significance of somatic mtDNA variants within (breast) cancer is still subject to debate, where the whole spectrum of neutral accumulation, positive selection (advantage) and negative selection (disadvantage) have been postulated. Various studies have shown that primary breast tumors harbor somatic variants in their mtDNA [8,15,16], with approximately 70% of the specimens containing at least one single nucleotide variant (SNV, range 1–7) and 10% containing at least one small insertion/deletion (INDEL, range 0–3). However, these variants do not appear at particular ‘hot-spot’ positions on the mitochondrial genome, raising doubts about their clinical relevance.

To better understand nucleotide changes in and expression of the mitochondrial genome within primary breast tumors, we investigated here transcriptomic sequencing data within the International Cancer Genome Consortium (ICGC) [17] and explored how these findings correlate with clinical parameters, providing more insight into the mitochondrial genome as potential biomarker and its clinical relevance in breast cancer.

## 2. Results

We evaluated RNA sequencing data of 344 primary breast tumor specimens. After mapping of sequencing reads against the human reference genome, median 15% (Interquartile range (IQR) 10–23%) of the uniquely mapped reads were assigned to the mitochondrial contig, resulting in median 9889× read depth (IQR 5333) of mtDNA.

### 2.1. Somatic Variants in mtRNA

**Figure 1:**
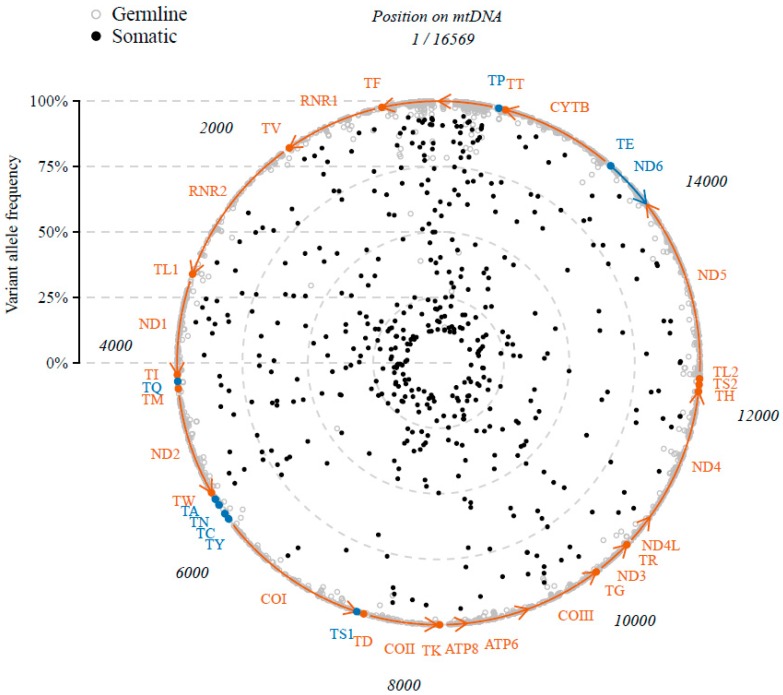
Variants in the mitochondrial RNA of 344 primary breast tumor cases. Position on the mitochondrial genome (circle) and their variant allele frequency (increasing % from inner-to-outer) of all variants identified in the 344 cases. Somatic or germline origin in respectively closed black or open grey circles. Genes and their direction of transcription (arrows) in red (+strand) or blue (−strand). Note that variants on position 2617 (known RNA-DNA differences) are not shown.

Variant calling resulted in a total of 9063 single nucleotide variants (SNVs) on 1600 positions and 84 small insertions or deletions (INDELs) on 38 positions of the mitochondrial genome within the 344 cases (Figure 1). Since INDELs were only a minority, our focus was on the SNVs only. We defined SNVs as somatically acquired tumor variants when not associated with the individual’s haplotype (*n* = 7235 excluded, 80%) or with heteroplasmic allele frequency of ≤95% (*n* = 917 excluded, 10%). Also, we defined the variants at position 2617 (r.2617a>u and r.2617a>g, present in respectively *n* = 340 and *n* = 101 cases) as not tumor-specific because (1) they have been described previously as RNA-DNA differences in blood cells of non-cancer patients [18,19] and (2) we confirmed their presence in a transcriptomic dataset of normal specimens of various tissue types including breast tissue [20] (Supplementary Materials Table S1). After these exclusions, a total of 470 somatic variants on 429 positions were identified.

**Figure 2:**
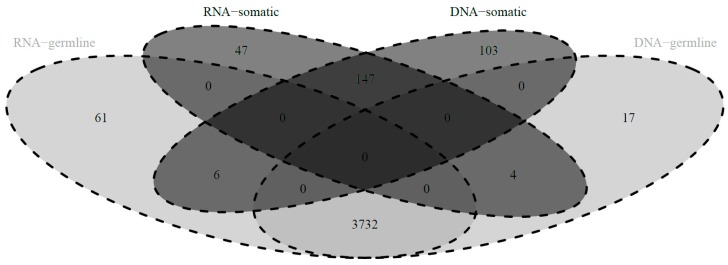
Classification of variants detected in the mitochondrial RNA and in the mitochondrial DNA of 165 primary breast tumor cases. Venn-diagram depicting classification of variants as either somatic (black) or germline (grey) at the RNA level and the DNA level.

Our dataset has overlapping cases (*n* = 165) with the dataset published by Ju et al. [15] concerning somatic mitochondrial variants in tumor and matched normal specimens at the DNA level. This allowed us to directly compare called variants between the two datasets (see also Appendix A) to evaluate presence, classification and allele frequency of variants. Since variants at position 2617 are known RNA-DNA differences (see above) and indeed not called in the DNA dataset, these were not included in this comparison. A total of respectively 3997 and 4009 SNVs were called at the RNA and DNA level within the primary tumor specimens of the 165 cases. The majority of the variants were called at both the RNA and DNA level (*n* = 3889, respectively 97.3% and 97.0%), whereas a small fraction was only called at either the RNA or the DNA level (respectively *n* = 108 (2.7%) and *n* = 120 (3.0%) variants) (Figure 2). Of the variants detected at both the RNA and DNA level, only a few (*n* = 10, 0.3%) had a discrepancy in classification as either ‘somatic’ or ‘germline’ (Figure 2). Also, good consistency was observed in allele frequency at the RNA and DNA level (linear fit coefficient of 0.92 for all variants and 0.96 for somatic tumor variants). From this we concluded that presence, classification and allele frequency of variants was consistent between the RNA and the DNA level (as elaborated on in the Appendix A).

**Figure 3:**
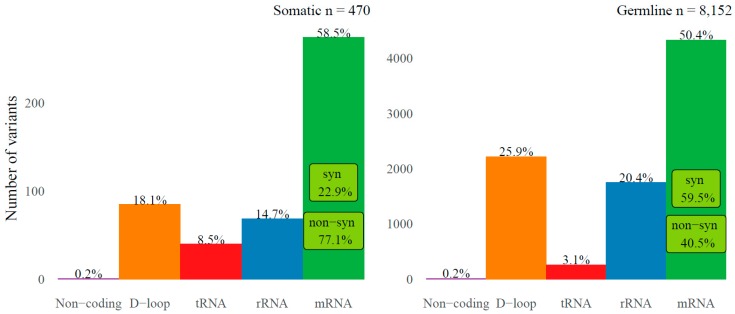
Genomic distribution of mitochondrial RNA variants of 344 primary breast tumor cases. Genomic distribution is depicted for somatic (**left**) or germline (**right**) variants in either non-coding (purple), the D-loop (orange), tRNA (red), rRNA (blue) or mRNA (green) regions of the mitochondrial genome. The percentage of total is indicated at the top of the bars. The percentage of substitutions in the mRNA regions with either a synonymous or nonsynonymous effect is indicated within the mRNA bar (light green). Note that variants at position 2617 (known RNA-DNA differences) are not included.

**Figure 4:**
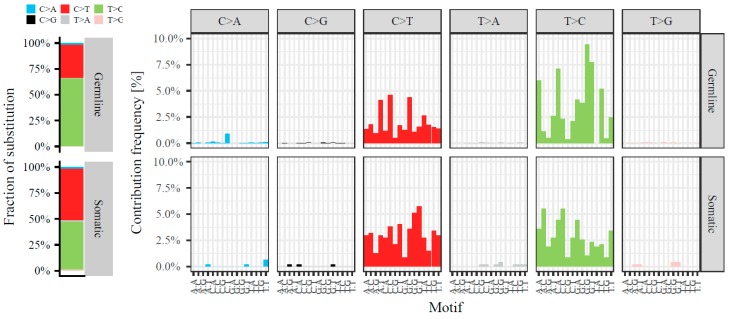
Somatic spectrum of mitochondrial RNA variants of 344 primary breast tumor cases. The contribution of the six possible base substitutions (C > A in blue, C > G in black, C > T in red, T > A in grey, T > C in green and T > G in pink) (**left**) and the context of each substitution (bases immediately 5′ and 3′ to each variant in the reference genome) (**right**) are depicted for the germline (top) and somatic (bottom) variants (left). Note that variants on position 2617 (known RNA-DNA differences) are not included.

We then continued to further decipher the somatic mtRNA variants in our dataset (*n* = 470 in *n* = 344 cases). The variant allele frequency of the somatic variants was distributed with a peak at the lower and at the upper end of allele frequencies (Supplementary Materials Figure S1). There was no correlation between the variant allele frequency and the percentage of invasive tumor cells in the evaluated specimen (Spearman correlation coefficient rho = 0.03, *p* = 0.5). The detected somatic variants were distributed along the entire mitochondrial genome (Figure 1), with 40 (8.5%) variants located in the tRNA genes, 69 (14.7%) in rRNA genes, 85 (18.1%) in the D-loop, 1 (0.2%) in the non-coding regions, and 275 (58.5%) in the mRNA genes of which 212 (77.1%) had a nonsynonymous effect on the coding amino acid (Figure 3). However, relative to their genomic size (9.0% tRNA genes, 15.1% rRNA genes, 6.8% D-loop, 0.4% non-coding and 68.7% mRNA genes) more variants were present in the D-loop and fewer in the mRNA genes (Fisher exact *p* < 0.001). Also in comparison to the germline variants (variants that were associated with the haplogroup of that individual or with an allele frequency > 95%, *n* = 8152) there was a difference in genomic distribution (Fisher’s exact *p* < 0.001) with fewer somatic variants in the D-loop but more in the tRNA and mRNA genes, and an enrichment for somatic nonsynonymous mRNA variants (Figure 3). The positions of somatic variants were much more conserved among species compared to the germline variants (Mann-Whitney test *p* < 0.001), as measured by the fraction of species that harbor the reference sequence at that position (Conservation Index of respectively median (IQR) 0.93 (0.36) and 0.76 (0.69)). A total of 69 (15%) somatic variants were recurrent and positioned on 28 mitochondrial positions. The majority of the somatic variants (95%) represented the typical replication-coupled mtDNA substitution pattern with predominantly C > T and T > C transitions as described previously [15,16,21] in a nucleotide context similar to the germline variants (Figure 4). However, compared to the detected germline variants the ratio between C > T and T > C variants is shifted (Fisher exact *p* < 0.001) with an increased number of C > T transitions among the somatic variants (Figure 4).

In the entire cohort, there are 112 (33%) cases with 0 somatic variants, 97 (28%) with 1 somatic variant, and 135 (39%) with more than 1 somatic variant (range 2 to 7). Of the cases with more than 1 somatic variant, 82 (61%) had a difference > 20% allele frequency between variants, indicative for (sub-)clonality.

### 2.2. Somatic Mitochondrial Variants in Relation to Somatic Variants in the Nuclear Genome

Next, to gain more insight into the relation between the mutational processes shaping mtDNA and nDNA, we associated the amount of somatic mtRNA variants with the number of somatic variants induced by the known major mutational patterns shaping the nDNA. For this purpose, we obtained for the overlapping cases (*n* = 268) the number of nDNA variants as published by Nik-Zainal et al. [17]. There was no statistically significant association between the number of somatic mtRNA variants and the total number of somatic variants in the nuclear DNA (Spearman correlation coefficient rho = 0.01, *p* = 0.8). Next, we combined per case the number of variants in nDNA associated with the mutational processes as described by Nik-Zainal et al. [17]: age-related (signatures 1 and 5), APOBEC-related (signatures 2 and 13) and homologous-recombination deficiency-related (signatures 3 and 8) processes. No statistically significant associations were observed between the number of somatic mtRNA variants and any of these three mutational processes (all Kruskal-Wallis *p* > 0.2). Note that only two samples within the dataset contained variants associated with mismatch-repair deficiency (signatures 6, 20 and 26), and none of samples contained variants associated with the signatures of unknown etiology (signatures 17, 18 and 30), as a consequence of which these specific subgroups could not be evaluated.

### 2.3. Mitochondrial Gene Expression

**Figure 5:**
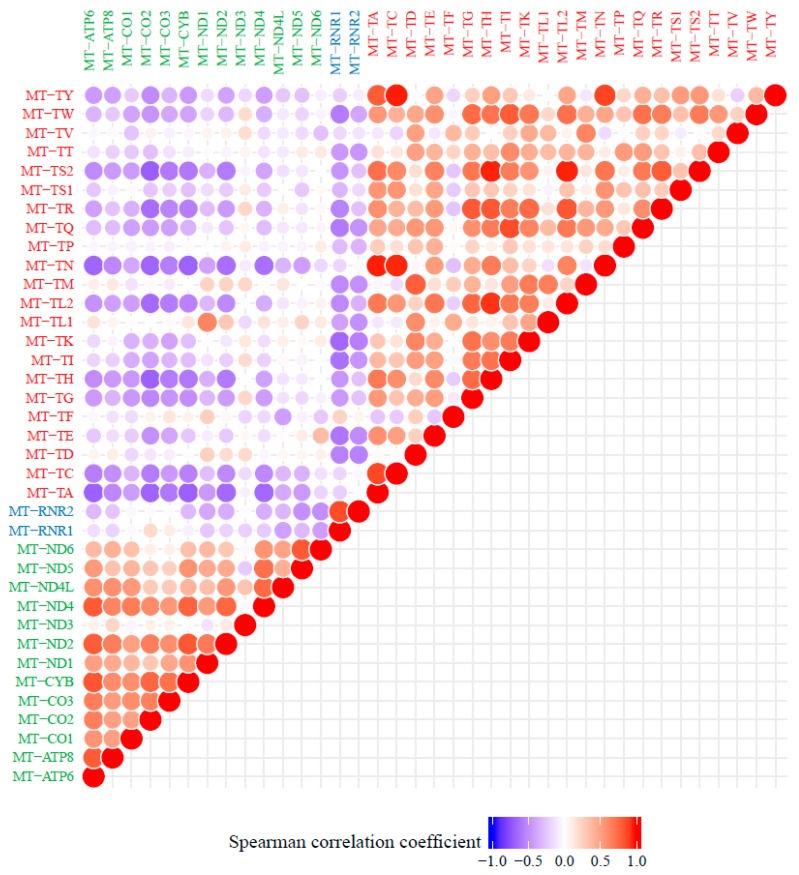
Correlation matrix of expression of all 37 mitochondrial-encoded genes of 344 primary breast tumor cases. Correlation matrix depicting the Spearman correlation between all 37 mitochondrial-encoded genes (text of tRNA genes in red, rRNA genes in blue, mRNA genes in green). Color intensity and the size of the circle are proportional to the correlation coefficients.

To estimate the expression and transcript processing of the mitochondrial genome for each case, transcripts per million (TPM, log2-transformed) were calculated for the entire mtDNA and each mitochondrial-encoded gene individually. Expression of the entire mtDNA—normalized against the nuclear genome and thus evaluated as driven by mtDNA content and transcription rate—was high and showed minor variability among the 344 cases (median 19.9210 TPM, IQR 0.0045). Within the 37 mitochondrial-encoded genes—normalized within the mitochondrial genome and thus evaluated as driven by processing of the polycistronic transcripts—the levels for genes encoding tRNAs were lowest (median 12.52 TPM, IQR 1.32), followed by mRNAs (median 15.37 TPM, IQR 0.31) and rRNAs (median 16.83 TPM, IQR 0.48). Most variability was observed in levels of tRNAs. Also, distinct correlation clusters were observed between the expression levels of the genes encoding mRNAs, tRNAs and rRNAs, where among genes a positive correlation was present per gene-type, but between different gene-types a negative correlation was present (Figure 5). No correlation was observed between the number of mtRNA variants and expression of the entire mtDNA (Spearman correlation coefficient rho = −0.02, *p* = 0.7).

### 2.4. Association with Clinicopathological Parameters

Lastly, we explored how these findings correlate with relevant clinical parameters. We analysed the number of somatic mtRNA variants (grouped variable as 0 variants, 1 variant and >1 variant per tumor, Table 1) and the expression of the entire mitochondrial contig (continuous variable, Table 1) in relation to traditional clinicopathological variables including age at diagnosis (*n* = 291 cases), tumor size (T-stage) (*n* = 216 cases), pathological grade (*n* = 282 cases), estrogen receptor (ER) status (*n* = 291 cases) and progesterone receptor (PR) status (*n* = 288 cases). Due to the low numbers of patients with HER2-amplified (*n* = 2 cases) and presenting with metastases at primary diagnosis (*n* = 3 cases), these clinicopathological variables were not evaluated. Age at diagnosis was statistically significant associated with both the number of somatic mtRNA variants (Kruskal-Wallis *p* = 0.022) and expression of the entire mtDNA (Spearman correlation coefficient rho = 0.11, *p* = 0.049), where a higher age corresponded to more somatic mtRNA variants and higher expression of the entire mtDNA. Also, a highly statistically significant association was observed between expression of the entire mtDNA and hormone receptor status (as evaluated at the protein level), with increased mtDNA expression in the ER-positive and in the PR-positive tumors (respectively Mann-Whitney U test *p* < 0.001 and *p* = 0.006). In fact, also a significant correlation was observed between expression of the entire mtDNA and RNA expression of *ESR1* or *PGR* (respectively Spearman correlation coefficient rho = 0.19 *p* < 0.001 and rho = 0.17 *p* = 0.001, *n* = 344 and *n* = 342 cases).

## 3. Discussion

In this work, we explored genomic changes in and expression of the mitochondrial genome within primary breast tumors, and their correlation with clinicopathological variables.

Within our breast tumor dataset, the fraction of reads mapping to the mitochondrial contig of the reference genome (median 15%) is in line with previous findings in non-tumorous breast samples: within the Illumina Body Tissue Atlas ~15% of the sequencing reads mapped to the mitochondrial genome (*n* = 1) [22], and within the Genotype-Tissue Expression (GTEx) Consortium ~15–20% of the transcriptional output was of mitochondrial origin (*n* = 27) [23]. This is in line with the requirement for functional mitochondria within cancer cells [24]. This also indicates that although the expression of the mitochondrial genome has been shown to be decreased in breast tumors compared to tumor-adjacent normal mammary tissue [11], the extent to which this occurs is less extreme than observed among tissue types (e.g., a much lower fraction of mitochondrial reads in blood (<5%) or much higher fraction in kidney (>50%) [23]). Nevertheless, we observed an association between expression of the entire mtDNA and ER status (measured at protein-level), with marginally higher expression in ER-positive tumors and a similar observation for PR status (protein-level) (Table 1). In addition, also RNA expression of *ESR1* and *PGR* was positively correlated with expression of the entire mitochondrial contig. The relation between expression of mtDNA and clinicopathological parameters has not been evaluated by others, but when we associated the data reported by Reznik et al. [11] on mtRNA expression within the TCGA-BRCA dataset (*n* = 656 cases) we observe a similar correlation for ER status (Kruskal-Wallis *p* = 0.006, Supplementary Materials Table S2 and none for the other clinicopathological variables (all *p* > 0.05 Supplementary Materials Table S2). In pre-clinical models, there appears to be a link between ER and mitochondrial activity: exposure to estrogens increases mitochondrial expression and oxygen consumption in ER-positive [25,26] but not in ER-negative breast cancer cells [26]. Similarly, ER-negative breast cancer cell lines show lower mitochondrial respiration and a stronger dependency on glycolysis in comparison to ER-positive breast cancer cells [27]. Unfortunately, measurements on mitochondrial activity comparing ER-positive and ER-negative clinical specimens are to our knowledge not reported in the literature, and thus the effect of differences in *ESR1* levels on mitochondrial activity in primary breast tumors remains currently unknown. Interestingly, uptake values of fluorodeoxyglucose (FDG) in positron emission tomography (PET)—a visualization of glucose uptake reflecting the increased rate of glycolysis in the tumor—appears to be higher in ER-negative cases [28,29,30,31,32,33,34], indicative that indeed metabolic differences are present between the subtypes. Additional studies should be performed to identify if there are differences in mitochondrial (oxidative phosphorylation) function among breast cancer subtypes and the potential clinical relevance of these findings, such as predictive and prognostic potential.

We also observed distinctive clustering of tRNA genes, which is in line with the tRNA punctuation model: when processing the polycistronic transcripts, tRNA genes are excised and due their small size (<75 base pairs) tRNAs are more likely to be lost during the RNA extraction and/or library preparation procedures, whereas the mRNA and rRNA genes are retained (>200 base pairs). Notably, we did not observe differences in this distinct pattern between the ER-positive and the ER-negative cases (Supplementary Materials Figures S2 and S3), and thus the processing of the polycistronic transcripts does not seem to differ between these two subtypes.

Our findings on the number, genomic distribution, and substitution pattern of mtDNA variants within the mitochondrial transcriptome are in line with previous studies on variants within the mitochondrial genome in other cancer types [8,15,16,21,35,36] (see also Appendix A). We observe an increased number of somatic variants in the D-loop and fewer in mRNA genes than expected by genomic size, which might be explained by the gene-dense constitution of mtDNA: variants in the D-loop potentially have less destructive effects whereas variants in the mRNA genes might have detrimental effects on the function of the oxidative phosphorylation system, and thus will be selected against. Also, the structural conformation of the D-loop (a triple-stranded structure) could make it more prone to damage. However, compared to germline variants in our dataset there are fewer variants in the D-loop and more in the tRNA and mRNA genes, and enrichment for nonsynonymous variants. This might be explained by the typical mutation pattern shaping mtDNA, which has been shaping the germline variants and thus the trivial positions have already been altered, as suggested by Ju et al. [15]. In line with this, the conservation of variants among species—the fraction of species that harbor the reference sequence at that position—was much higher for somatic variants than for the germline variants, which can be explained by the same hypothesis. Adding to this, compared to the detected germline variants there is an increased number of C > T transitions among the somatic variants (Figure 4). Note that the functional effect of somatic mtDNA variants on mitochondrial function is dependent on the actual position (e.g., protein-coding regions) and consequence (e.g., stop-gain or nonsynonymous) of the variant in combination with their heteroplasmy level within the tumor cell, rather than merely the number of somatic variants observed.Adjusting variant allele frequency to account for sample purity (percentage of tumor cells within the specimen) is often applied for nuclear-encoded genes to obtain information on the allele frequency of variants in the tumor cells. However, this is not possible for mtDNA variants in tumor tissue specimens: the number of mtDNA molecules per cell largely varies among cell types and thus the non-tumor cells present in the specimen do not have the regular two copies as the nuclear genome would have, but contain multiple mtDNA copies of an unknown number. As a result, whereas allele frequency of variants could give information on possible constraints on variants, we did not perform analysis on it since it is impossible to estimate the actual allele frequency of variants in the mitochondria of tumor cells. Nevertheless, we show that majority of the samples with more than 1 somatic variant harbor a difference in variant allele frequency between variants, indicative for (sub-)clonality. This corresponds to the hypothesis that mtDNA variants are either expanded or lost [37] and that the mutations occur separated in time [15].

Also noteworthy is that with the current methodologies applied by us and by others—namely the use of non-micro dissected tumor specimens and blood as matched normal DNA—we cannot be completely sure that the detected somatic mtDNA mutations are tumor-specific. First, tumor tissue specimens consist of multiple cell types, including the tumor cells but also non-neoplastic cells such as immune cells and cells from the mammary epithelium, all with variable mtDNA content. Secondly, (somatic) mtDNA variant heteroplasmy patterns can differ within an individual across tissues [38,39,40,41]. Thus, the somatic variants were either acquired in the tumor, the normal somatic epithelium, or even in other cell types present within the specimen.

We did not observe associations between the number of somatic mtRNA variants and the three major mutational processes shaping the nDNA within breast tumors. This is in line with the hypothesis that mutations within the mitochondrial genome are mainly due to fidelity of the mitochondrial polymerase [42] and thereby hardly due to exogenous factors [15]. Accordingly, in our evaluation of associations with clinicopathological parameters we observed a statistically significant association between the number of mtRNA somatic variants and age at diagnosis. Previous work on somatic variants at the DNA level also revealed a correlation with older age of diagnosis (*n* = 381 [15] and *n* = 58 cases [35]). Previous work in a small cohort also showed associations between number of somatic variants in mtDNA and higher TNM and higher histological grade (*n* = 58 cases [35]), which we did not observe. Please note that there are differences in the composition of the cohorts; our dataset does not exactly represent the breast cancer population as seen in daily practice, with an underrepresentation of *ERBB2*-amplified cases (Supplementary Materials Table S3).

By using data at the RNA level, we intended to minimize the interference of NUMTs with evaluation of mtDNA expression and variant calling, since their expression in the nucleus is negligibly low [11,43]. Especially in defining heteroplasmic mtDNA variants in DNA data, NUMTs have been shown to be a complicating issue with non-identical positions misinterpreted as heteroplasmic variants [44,45,46,47,48]. Note that we do observe a few heteroplasmic variants at the DNA-only level (Appendix A). However, using data at the RNA level comes with the trade-off that only variants in expressed regions are detected and thus variants in non-expressed regions are missed. Since mtDNA is a gene-dense entity, we estimate that the number of missed variants should be low. Indeed, in our direct comparison of samples with variants at the RNA and DNA level, we show that this is maximally ~3% of the variants (DNA-only variants). Similar to these findings, the comparison by Stewart et al. [16] on somatic variants at the RNA and DNA level showed 7 of the 130 variants (5%) detected at only the DNA level within their set of 100 breast cancer specimens. Another trade-off using RNA is the additional step to generate cDNA, which might induce false positive calls by mistakes of the reverse transcriptase. Again based on our direct comparison of samples with variants at the RNA and DNA level, the number of false positives is maximally 3% of the detected variants (RNA-only variants). Though, besides false positives, these RNA-only variants might actually be RNA-DNA differences for example caused by RNA-editing [49], or true variants not called at the DNA level.

## 4. Materials and Methods

### 4.1. Data

We studied all patients with RNA sequencing data within the ICGC BASIS consortium, of which the cohort has been described previously [17] and data deposited in the European-Genome Phenome Archive (accession code EGAS00001001178). Briefly, for a total of 348 primary breast tumors we generated duplex-specific nuclease-based RNA sequencing data. Four samples were excluded from analyses due to potential cross-contamination (see below). We did not apply a threshold on tumor cell percentage within the specimen for inclusion in this study. Clinicopathological data and the nuclear somatic mutation catalogue were obtained from the Supplementary Tables as provided by Nik-Zainal et al. [17]. Expression levels of *ESR1*, *PGR* (quantile normalized FPKM, log2 transformed) were obtained as described previously [50]. A complete dataset on all variables used in our analyses is provided in Supplementary Materials Table S3. In addition, we used publically available RNA sequencing data of twelve human tissue specimens obtained via a similar sequencing approach [20], that has been deposited in NCBI’s Gene Expression Omnibus (GEO) (accession code GSE45326). Also, we used the mtDNA variants called by Ju et al. [15] from whole-genome or whole-exome sequencing data of DNA from the primary breast tumor specimens and matched normal tissue specimens as provided in their Supplementary Tables.

### 4.2. Bioinformatics

Sequencing reads were aligned using STAR v2.4.2.a [51] against the Genome Reference Consortium Human Build 38 (GRCh38, GenBank assembly GCA_000001405.15), which contains as the mitochondrial contig the revised Cambridge Reference Sequence (rCRS). Only non-duplicated uniquely mapped reads on mtDNA were used for further analysis, to avoid the potential use of improper assigned nuclear insertions of mitochondrial origin (NUMTs, mitochondrial pseudogenes). Note that RNA expression of NUMTs has been shown to be absent or negligibly low [11,43]. Total read depth was estimated based on the read length (75 nucleotides) and mtDNA size (16,569 nucleotides). FeatureCounts v 1.4.6 [52] was used to count mapped reads using mtDNA as the meta-feature and each genomic region (13 mRNAs, 22 tRNAs, 2 rRNAs) as the features, allowing multi-overlapping reads (-O) because of the polycistronic nature of mitochondrial RNA transcripts. We normalized read counts to transcripts per million (TPM) for the entire mitochondrial contig (mtDNA read counts versus total read counts assigned to genes in GRCh38, defined as entire mtDNA levels) and for each mitochondrial-encoded gene (gene read counts versus total mtDNA read counts, defined as <*gene*> levels). In this way, the TPM for the entire mtDNA represents the total amount of mtRNA influenced by both mtDNA content, transcription rate and transcript stability, whereas the TPM for each mitochondrial-encoded gene represents the variation in gene expression driven by processing of the polycistronic transcripts and transcript stability [53]. A complete dataset of all expression levels is provided in Supplementary Materials Table S4. Variants alternative to rCRS were called using GATK HaplotypeCaller 3.4-46-gbc02625 [54] using default settings (including downsampling_type = BY_SAMPLE, downsample_to_coverage = 500, standard_min_confidence_ threshold_for_calling = 20). In this way, maximum depth of coverage is controlled at each locus, resulting in a more even coverage of variants between the samples. Hard-filtering was applied to the called variants for quality by depth (QD > 2), alternative depth (AD of ALT > 10) and strand odds ratio (variants with allele frequency ≤ 95% i.e., heteroplasmic variants: SOD < 4 for SNVs and SOD < 10 for INDELs; variants with allele frequency > 95% i.e., (near) homoplasmic: no filtering). In this way, the allele frequency of detected variants was high and confident enough to be a true variant and likely no sequencing errors or PCR mistakes. In addition, after visual inspection of variants (Integrative Genomics Viewer [55,56]), potential false positive calls in challenging regions were excluded: positions surrounding the homopolymer region 301–315 (“D310”), positions 512–513 due to a repetitive sequence, alternative C calls at positions 16,182–16,183 and 16,189 due to polyC sequences, and alternative A at positions 4264, 5513 and 12,138–12,139 due to polyA sequences. A complete dataset of all remaining variants is provided in Supplementary Materials Table S5. All remaining single nucleotide variants were used in a nucleotide BLAST against the human reference sequence (NCBI’s nucleotide web blast, https://blast.ncbi.nlm.nih.gov) with the surrounding reference sequence (30 bases 5′ and 30 bases 3′) to uncover potential NUMT events, but none were recovered. The conservation index (45 species conservation) for the protein-coding genes, tRNAs and rRNAs were obtained via SNV Query in Mitomaster [57]. The haplotype of each case was estimated by using the heteroplasmic and homoplasmic variants in HaploGrep v2 [58]. Sample cross-contamination was estimated using only the heteroplasmic variants (allele frequency ≤ 95%) in haplotype assignment. This identified four samples with heteroplasmic contamination of another haplotype, therefore these samples were excluded from analyses. Sample mismatch between cases with variants called in both RNA (our dataset) and DNA (dataset Ju et al. [15]) sequencing data (*n* = 168) was estimated by haplotyping based on all near-homoplasmic variants (allele frequency > 95%), and comparison of the obtained haplogroup. Mismatch was observed for 13 patients, but after manual inspection specificity could be confirmed for 10 patients by the presence of private variants. Two patients with a clear mismatch, and one patient ambiguous in mismatch, were excluded from the RNA-DNA comparison analyses (*n* = 165 remaining).

### 4.3. Statistics

Performed statistical tests are reported in the results section. All statistical tests were two-sided, and P values smaller than 0.05 were considered as statistically significant. Outliers data points in boxplots are defined as Q1−1.5*IQR or Q3+1.5*IQR. Analyses were performed in R version 3.3.2 (https://cran.r-project.org). Data analyses included usage of the following packages: the set of tidyverse, ggcorplot, SomaticSignatures [59] and VennDiagram [60].

## 5. Conclusions

To conclude, in this explorative study on the role of mtRNA in breast cancer, we found that somatic variants at the DNA level are reflected at the RNA level with no hotspot mutations and great heterogeneity across tumors. We confirm that the number of somatic variants within the mitochondrial transcriptome is not associated with the mutational processes shaping the nuclear genome but instead, is associated with age of diagnosis. Furthermore, we show that mitochondrial expression is related to ER status. The exact consequence of the observed differences in mtRNA expression and the detected somatic variants on cancer metabolism and clinical outcome warrants further study.

## Figures and Tables

**Table 1:** Association between number of somatic tumor mtRNA variants or expression of the entire mtDNA and clinicopathological variables.

Variable	No. of Cases	mtRNA Somatic Variants			*p*	mtRNA Expression	*p*
		0 Variants	1 Variant	>1 Variants		Median (IQR) TPM	
Age					0.022 ^a^		0.049 ^d^
56 (28–85)	291 (100%)	53 (17)	55 (23)	61 (24)		0.11 ^c^	
unknown	*53*						
Tumor size					0.07 ^b^		0.051 ^a^
T1 ≤ 2 cm	76 (35.2%)	33.8%	25.0%	44.4%		19.9202 (0.0043)	
T2 > 2–5 cm	109 (50.5%)	47.9%	64.1%	42.0%		19.9207 (0.0045)	
T3 > 5 cm	31 (14.4%)	18.3%	10.9%	13.6%		19.9223 (0.0047)	
unknown	*128*						
Grade					0.4 ^b^		0.1 ^a^
I	24 (8.5%)	9.9%	12.2%	5.1%		19.9202 (0.0037)	
II	111 (39.4%)	40.7%	35.1%	41.0%		19.9216 (0.0044)	
III	147 (52.1%)	49.5%	52.7%	53.8%		19.9209 (0.0049)	
unknown	*62*						
ER					0.3 ^b^		<0.001 ^a^
Negative	81 (27.8%)	21.7%	31.2%	30.3%		19.9196 (0.0050)	
Positive	210 (72.2%)	78.3%	68.8%	69.7%		19.9216 (0.0041)	
unknown	*53*						
PR					0.5 ^b^		0.006 ^a^
Negative	102 (35.4%)	31.5%	40.5%	35.0%		19.9204 (0.0048)	
Positive	186 (64.6%)	68.5%	59.5%	65.0%		19.9215 (0.0042)	
unknown	*56*						

For each subgroup within the clinicopathological variable, the number of cases and either the fraction of patients within the mtRNA somatic variant groups (0, 1 or more than 1) or the mtRNA expression (TPM, log2 transformed) is indicated. ^a^ Kruskal-Wallis (multiple groups) or Mann-Whitney (two groups) *p*-value. ^b^ Fisher exact *p*-value. ^c^ Spearman correlation coefficient. ^d^ Spearman correlation *p*-value.

**Figure A1:**
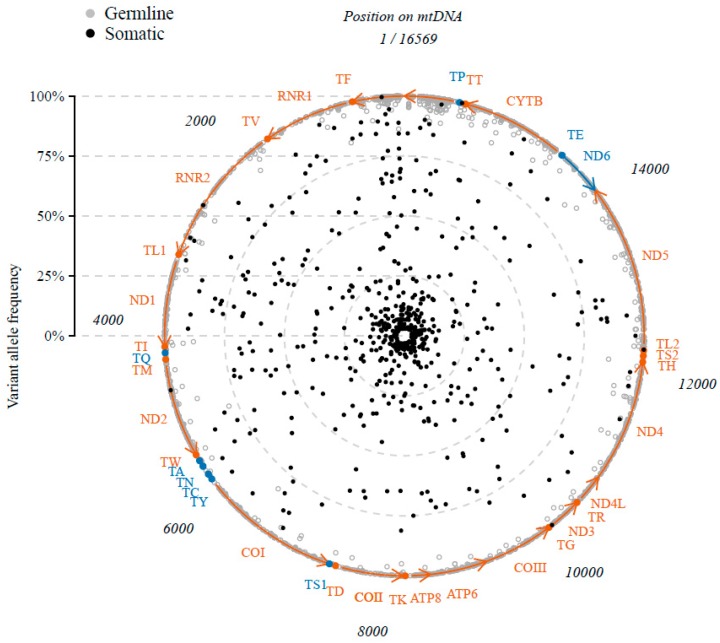
Variants in the mitochondrial DNA of 381 primary breast tumor cases. Position on the mitochondrial genome (circle) and their variant allele frequency (increasing % from inner-to-outer) of all variants identified in the 381 cases. Somatic or germline origin in respectively closed black or open grey circles. Genes and their direction of transcription (arrows) in red (+strand) or blue (−strand).

**Figure A2:**
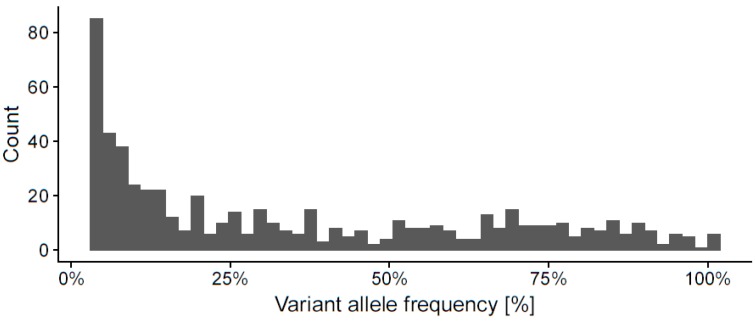
Histogram of variant allele frequency of the somatic mitochondrial DNA variants of 381 primary breast tumor cases. Histogram of the variant allele frequency (horizontal) of the somatic mitochondrial DNA variants detected in the 381 primary breast tumor cases.

**Figure A3:**
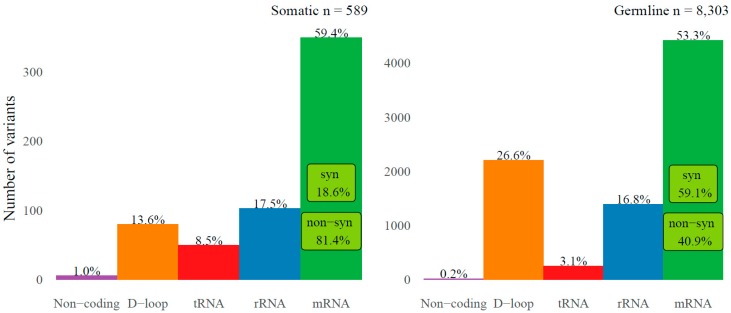
Genomic distribution of mitochondrial DNA variants of 381 primary breast tumor cases. Genomic distribution is depicted for somatic (left) or germline (right) variants in either non-coding (purple), the D-loop (orange), tRNA (red), rRNA (blue) or mRNA (green) regions of the mitochondrial genome. The percentage of total is indicated at the top of the bars. The percentage of substitutions in the mRNA regions with either a synonymous or nonsynonymous effect is indicated within the mRNA bar (light green).

**Figure A4:**
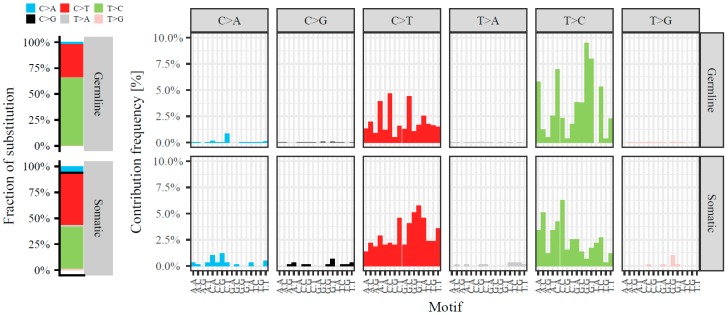
Somatic spectrum of mitochondrial DNA variants of 381 primary breast tumor cases. The contribution of the six possible base substitutions (C > A in blue, C > G in black, C > T in red, T > A in grey, T > C in green and T > G in pink) (**left**) and the context of each substitution (bases immediately 5′ and 3′ to each variant in the reference genome) (**right**) are depicted for the germline (top) and somatic (bottom) variants (left).

**Figure A5:**
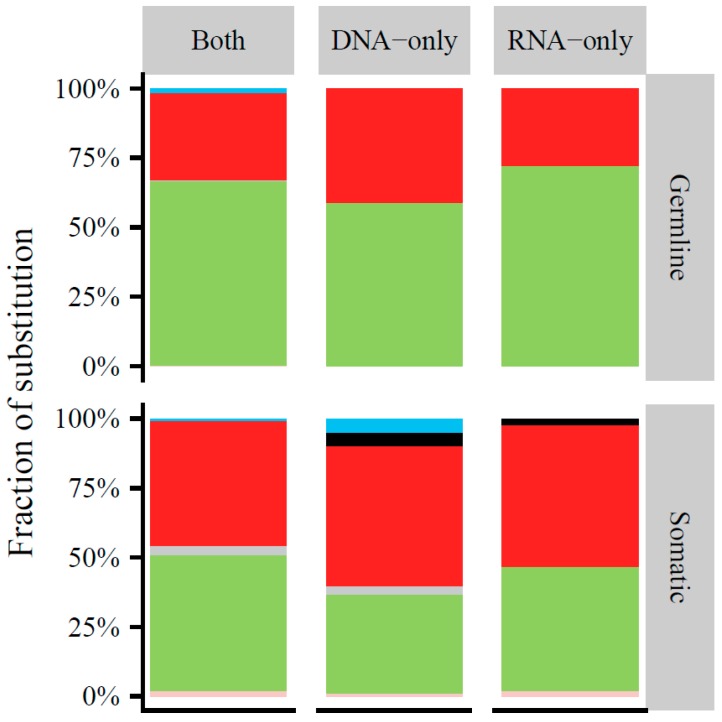
Somatic spectrum of mitochondrial DNA and mitochondrial RNA variants of the 165 overlapping primary breast tumor cases. The contribution of the six possible base substitutions (C > A in blue, C > G in black, C > T in red, T > A in grey, T > C in green and T > G in pink) are depicted for the germline (top) and somatic (bottom) variants detected in both (left) the DNA (middle) or the RNA (right) datasets.

## Supplementary Materials

The following are available online at http://www.mdpi.com/2072-6694/10/12/500/s1, Figure S1: Histogram of variant allele frequency of the somatic mitochondrial RNA variants of 344 primary breast tumor cases, Figure S2: Correlation matrix of expression of all 37 mitochondrial-encoded genes of 81 ER-negative primary breast tumor cases, Figure S3: Correlation matrix of expression of all 37 mitochondrial-encoded genes of 210 ER-positive primary breast tumor cases, Table S1: Position 2617 in normal tissue specimens, Table S2: Association between mtRNA expression and clinicopathological variables in TCGA-BRCA, Table S3: Clinicopathological and other variables per sample (*n* = 344 cases), Table S4: Expression levels of mtRNA per sample (*n* = 344 cases), Table S5: Variants in mtRNA per sample (*n* = 344 cases).

## Appendix A

### Appendix A.1. Comparison of Mitochondrial Variants at the RNA and DNA Level

By using the dataset published by Ju et al. [15] concerning somatic mitochondrial variants in tumor and matched normal specimens at the DNA level, we intended to compare mitochondrial variants called in primary breast tumor tissue specimens at the RNA and the DNA level. Their dataset includes a total of *n* = 381 breast tumor specimens, of which *n* = 165 cases are overlapping with our dataset.

The DNA dataset contains 8892 single nucleotide variants (SNVs) on 1744 positions within the 381 cases (Figure A1), of which 589 variants classified as somatic (using VarScan2, see Ju et al. [15]).

The variant allele frequency of these somatic variants was distributed with a peak at the lower end of allele frequencies (Figure A2). The detected somatic variants were distributed along the entire mitochondrial genome (Figure A1), with 50 (8.5%) variants located in the tRNA genes, 103 (17.5%) in rRNA genes, 80 (13.6%) in the D-loop, 6 (1.0%) in the non-coding regions, and 350 (59.4%) in the mRNA genes of which 285 (81.4%) had a nonsynonymous effect on the coding amino acid (Figure A3). Relative to their genomic size (9.0% tRNA genes, 15.1% rRNA genes, 6.8% D-loop, 0.4% non-coding, 68.7% mRNA genes) more variants were present in the D-loop and fewer in the mRNA genes (Fisher exact *p* < 0.001). Also compared to the germline variants, there is a difference in genomic distribution: fewer somatic variants in the D-loop but more in the tRNA and mRNA genes, and an enrichment for somatic nonsynonymous mRNA variants (Fisher exact *p* < 0.001) (Figure A3). The positions of somatic variants were much more conserved among species compared to the germline variants (Mann-Whitney test *p* < 0.001), as measured by the fraction of species that harbor the reference sequence at that position (Conservation Index of respectively median (IQR) 0.96 (0.36) and 0.76 (0.69)).

A total of 74 (12.6%) somatic variants were recurrent and positioned on 34 mitochondrial positions. Also, majority of the somatic variants (89.5%) represented the typical replication-coupled mtDNA substitution pattern with predominantly C > T and T > C transitions in a nucleotide context similar to the germline variants (Figure A4). Compared to the detected germline variants the ratio between C > T and T > C variants is shifted (Fisher exact *p* < 0.001) with an increased number of C > T transitions among the somatic variants (Figure A4). In the cohort, there are 101 (26%) cases with 0 somatic variants, 117 (31%) with 1 somatic variant, and 163 (43%) with more than 1 somatic variant (range 2 to 7). Of the cases with more than 1 somatic variant, 103 (63%) had a difference >20% allele frequency between variants, indicative for (sub-)clonality.

When comparing these findings at the DNA level with our findings at the RNA level, no differences were observed in genomic distribution of the somatic variants (Fisher’s exact *p* = 0.1), the conservation index of somatic variants (Mann-Whitney test *p* = 0.4), and the recurrence rate (Fisher’s exact *p* = 0.3). However, the substitutional pattern differed, with a higher fraction of C > A substitutions at the DNA level (4.8%) compared to the RNA level (1.1%) (Fisher’s exact *p* < 0.001).

### Appendix A.2. Direct Comparison of Mitochondrial Variants at the RNA and DNA Level of Overlapping Cases

We next focused on the variants called within the overlapping cases within our RNA and the published DNA dataset (*n* = 165 cases). As stated in the main manuscript text, of the variants detected at both the RNA and DNA level only a few (*n* = 10, 0.3%) had a discrepancy in classification as either ‘somatic’ or ‘germline’ (resp. *n* = 4 and *n* = 6, Table A1). These were misclassifications at the RNA level, mainly due to the absence of information on the matched normal tissue: variants misclassified as ‘germline’ at the RNA level had allele frequencies > 95%, indicative for germline origin, but were not detected in the matched normal DNA whereas they were present in the matched tumor DNA and thus of somatic origin. Also, variants misclassified as ‘somatic’ at the RNA level had allele frequencies between 85% and 95% allele frequency, but were detected in the matched normal DNA as well as the matched tumor DNA and thus of germline origin. Also, of the variants detected at both the RNA and DNA level, only a few variants (*n* = 7, 0.2%) showed a strong deviation in variant frequency (>30% difference) (*n* = 3 germline and *n* = 4 somatic) (Table A2). In contrast to previous observations that mainly variants in tRNAs have allelic imbalances [9], none of them occurred at tRNA sites.

We continued evaluating the variants called within the overlapping cases present at either only the DNA or only the RNA level. A total of 120 variants (3.0%) were only called at the DNA level (103 somatic and 17 germline) (Table A2) and 108 variants (2.7%) were present at only the RNA level (47 somatic and 61 germline) (Table A3). Within the aligned reads of the RNA data (BAM file) we inspected if variants called at the DNA-only level were truly not present at the RNA level or just not called (Table A2). Majority of the called DNA variants were present in the RNA alignment data but not called by our used algorithm (*n* = 108, 90%), a few variants were not (sufficiently) covered (*n* = 5, 4%), and some were sufficiently covered but truly not present as alternative allele (*n* = 7, 6%). Unfortunately, we were unable to visually inspect variants in the DNA alignment data (not available) and thus the relevance of variants present at only the RNA level was not evaluable. Interestingly, when evaluating the substitution pattern of variants detected at both the RNA and DNA level (Figure A5), and at either the RNA or DNA level, the higher fraction of C > A substitutions at the DNA level compared to the RNA level appeared mainly due to variants called at only the DNA level.

Given these results, the differences we observe in called variants at the RNA and DNA level is likely an effect of differences in either the expression at the RNA level (biological) the calling algorithms used (technical).

**Table A1:** Positions misclassified at RNA versus DNA.

Sample	Variant	Depth RNA Tumor	VAF RNA Tumor	Class RNA	Variant	Depth DNA Tumor	VAF DNA Tumor	Depth DNA Normal	VAF DNA Normal	Class DNA
P_6042a	r.199u>c	1953	99.3%	Germline	g.199T>C	6585	67.1%	208	0.5%	Somatic
P_9571a	r.1010a>c	1655	97.1%	Germline	g.1010A>C	21,840	94.9%	763	0.0%	Somatic
P_6409a	r.4344u>c	146	97.9%	Germline	g.4344T>C	16,068	68.9%	1707	0.1%	Somatic
P_6043a	r.5353g>a	1099	97.6%	Germline	g.5353G>A	20,514	88.0%	544	0.0%	Somatic
P_5956a	r.11453g>a	1655	96.6%	Germline	g.11453G>A	21,214	96.3%	4230	0.0%	Somatic
P_9568a	r.14841a>g	1877	97.4%	Germline	g.14841A>G	12,060	86.5%	806	0.1%	Somatic
P_4982a	r.94g>a	698	91.1%	Somatic	g.94G>A	9643	99.8%	1449	99.5%	Germline
P_4982a	r.152u>c	614	93.0%	Somatic	g.152T>C	11,738	99.8%	2090	99.0%	Germline
P_8622a	r.497c>u	39	84.6%	Somatic	g.497C>T	11,020	99.8%	1975	99.5%	Germline
P_4963a	r.16302a>g	708	94.7%	Somatic	g.16302A>G	7042	97.3%	7860	86.6%	Germline

**Table A2:** Variants called at only the mtDNA level.

Sample	Variant	Gene	Depth DNA Tumor	VAF DNA Tumor	Depth DNA Normal	VAF DNA Normal	Depth RNA Tumor	VAF RNA Tumor	Concordant?
P_6719a	g.2A>T	Control-Region	81	4.94	59	0	15	0.00	*na*
P_6409a	g.66G>T	Control-Region	1712	3.27	465	0	644	0.16	*na*
P_9569a	g.73A>G	Control-Region	6118	11.43	146	0	1010	11.58	Yes
P_6719a	g.185G>A	Control-Region	3193	99.72	3421	99.18	10	100.00	Yes
P_9592a	g.195T>C	Control-Region	7012	4.04	2672	0.04	1076	3.35	Yes
P_4977a	g.263A>G	Control-Region	10,036	100	2142	99.91	9	100.00	Yes
P_9597a	g.293T>C	Control-Region	2568	12.27	810	0	1503	7.25	Yes
P_4847a	g.307C>A	Control-Region	1275	22.98	2124	0.09	228	0.00	No
P_4958a	g.316G>A	Control-Region	3513	10.65	698	2.72	376	3.99	Yes
P_5947a	g.319T>C	Control-Region	2942	4.11	1185	0.25	1015	26.21	Yes
P_5947a	g.321T>C	Control-Region	3184	4.74	1227	0.08	1017	26.16	Yes
P_4847a	g.346T>C	Control-Region	1557	31.34	2300	0.04	304	37.17	Yes
P_4847a	g.347G>A	Control-Region	1492	31.37	2030	0.05	305	35.08	Yes
P_11340a	g.456C>T	Control-Region	8351	99.8	462	99.78	7	100.00	Yes
P_9571a	g.462C>T	Control-Region	17,653	99.93	584	99.83	0	-	Yes
P_6719a	g.462C>T	Control-Region	5300	99.87	5718	99.88	4	100.00	Yes
P_4069a	g.462C>T	Control-Region	9108	99.89	1760	100	21	95.24	Yes
P_9571a	g.489T>C	Control-Region	18,133	99.98	619	100	2	100.00	Yes
P_6719a	g.489T>C	Control-Region	5798	99.91	5977	99.97	4	100.00	Yes
P_4069a	g.489T>C	Control-Region	9408	99.97	1766	100	18	100.00	Yes
P_6422a	g.549C>T	Control-Region	15,224	99.76	9454	99.27	15	93.33	Yes
P_5928a	g.730A>T	MT-RNR1	12,334	12.93	395	0	5056	4.94	Yes
P_11389a	g.903T>C	MT-RNR1	11,568	4.76	1006	0	5053	0.26	Yes
P_6413a	g.1284T>C	MT-RNR1	8416	25.5	2589	0.04	5030	1.83	Yes
P_11399a	g.1320G>A	MT-RNR1	6173	4.47	1995	0	5039	0.02	No*
P_4845a	g.1464G>A	MT-RNR1	6496	12.96	7902	0.19	5018	6.50	Yes
P_6719a	g.1748G>A	MT-RNR2	10,396	25.31	9000	0.06	4674	10.68	Yes
P_9754a	g.1758T>C	MT-RNR2	10,021	3.42	2541	0.04	5035	1.81	Yes
P_8618a	g.1906G>C	MT-RNR2	8094	3.43	5953	0.03	5038	0.02	No*
P_11384a	g.1913G>A	MT-RNR2	8494	4.07	2090	0.05	5031	0.58	Yes
P_9592a	g.1939G>A	MT-RNR2	16,324	3.59	6432	0.06	5022	2.59	Yes
P_6413a	g.1987G>A	MT-RNR2	8817	11.47	2498	0.04	4972	1.57	Yes
P_11380a	g.2024C>T	MT-RNR2	12,996	14.06	647	0	5024	2.31	Yes
P_11377a	g.2343G>A	MT-RNR2	7852	3.48	1118	0	5049	0.04	No*
P_9572a	g.2492G>A	MT-RNR2	13,639	3.43	671	0.15	5049	5.27	Yes
P_7221a	g.2571G>A	MT-RNR2	8410	3.22	2488	0.08	5007	3.40	Yes
P_11374a	g.2695G>A	MT-RNR2	4771	3.98	1416	0	5045	0.71	Yes
P_4976a	g.2716G>A	MT-RNR2	21,489	7.3	18,243	0.06	4996	4.06	Yes
P_5950a	g.3065T>C	MT-RNR2	7371	5.24	1548	0	4870	3.72	Yes
P_8980a	g.3068G>A	MT-RNR2	15,125	12.65	3374	0.12	4997	4.16	Yes
P_9573a	g.3097T>C	MT-RNR2	10,249	9.23	592	0.17	5036	5.90	Yes
P_7215a	g.3617T>C	MT-ND1	12,614	3.24	2196	0	1400	4.07	Yes
P_11375a	g.3715G>C	MT-ND1	9098	3.7	339	0.59	4889	1.82	Yes
P_4080a	g.4153G>A	MT-ND1	14,450	7.7	2259	0.04	53	3.77	Yes
P_6411a	g.4308G>A	MT-TI	13,780	3.72	1097	0	738	6.91	Yes
P_7218a	g.4336T>C	MT-TQ	11,432	99.9	2181	100	3	66.67	Yes
P_8979a	g.4399T>C	MT-TQ	7338	20.37	1572	0	29	20.69	Yes
P_4833a	g.4412G>A	MT-TM	10,532	5.86	4078	0.02	1039	87.20	Yes
P_4072a	g.4429G>A	MT-TM	16,360	55.94	2245	0.04	548	85.22	Yes
P_9777a	g.4582T>C	MT-ND2	3336	17.99	2439	0	19	0.00	*na*
P_11819a	g.4924G>A	MT-ND2	7357	9.11	4036	0.05	3760	6.97	Yes
P_4080a	g.5581A>G	Non-Coding	17,806	99.66	2669	99.96	0	-	Yes
P_6728a	g.5582A>G	Non-Coding	13,659	99.34	11,657	99.91	9	100.00	Yes
P_4982a	g.5703G>A	MT-TN	13,401	91.4	2276	0.04	1963	89.61	Yes
P_4971a	g.5920G>A	MT-CO1	6202	3.58	1517	0.07	4376	7.24	Yes
P_11340a	g.6255G>A	MT-CO1	11,906	6.16	824	0	1638	9.77	Yes
P_6422a	g.6673T>C	MT-CO1	20,090	4.39	12,278	0.02	1080	5.28	Yes
P_9574a	g.6724T>C	MT-CO1	10,549	6.03	871	0	5018	6.10	Yes
P_9574a	g.7191T>C	MT-CO1	11,204	6.12	932	0.11	5024	5.77	Yes
P_11377a	g.7207G>A	MT-CO1	8421	5.85	1093	0	5036	2.76	Yes
P_7214a	g.7219G>A	MT-CO1	11,168	3.36	2565	0	4983	3.45	Yes
P_4080a	g.7595G>A	MT-CO2	17,880	20.37	2789	0.07	3	0.00	*na*
P_7219a	g.7652T>C	MT-CO2	9000	7.7	2654	0.04	4787	7.65	Yes
P_11336a	g.7935T>C	MT-CO2	9586	5.42	594	0.34	4985	7.36	Yes
P_9592a	g.8213G>A	MT-CO2	15,889	3.63	6009	0.02	4964	3.28	Yes
P_4967a	g.8249G>A	MT-CO2	13,941	3.21	7740	0.03	5031	0.85	Yes
P_9572a	g.8269G>C	MT-CO2	6539	3.17	404	1.24	4858	0.00	No
P_11372a	g.8270C>T	Non-Coding	1447	98.48	184	96.2	4755	99.75	Yes
P_9002a	g.8278C>G	Non-Coding	2694	15.55	75	6.67	3092	0.00	No
P_9572a	g.8290G>C	Non-Coding	6059	5.73	370	2.43	96	1.04	*na*
P_9572a	g.8291A>C	Non-Coding	6217	4.68	382	2.36	93	5.38	Yes
P_3989a	g.8448T>C	MT-ATP8	11,980	8.68	5300	0.02	5042	1.23	Yes
P_7214a	g.8547T>C	MT-ATP8/6	7924	3.14	1835	0.05	3949	0.20	Yes
P_4977a	g.8860A>G	MT-ATP6	13,075	99.99	3626	99.97	46	97.83	Yes
P_9754a	g.9053G>A	MT-ATP6	10,819	4.92	2532	0	5011	3.47	Yes
P_9001a	g.9078T>C	MT-ATP6	14,729	5.89	497	0.2	2045	3.03	Yes
P_4958a	g.9181A>G	MT-ATP6	9010	39.35	2221	0	4870	49.96	Yes
P_5936a	g.9285A>T	MT-CO3	6732	11.51	3408	0.03	4916	6.96	Yes
P_6413a	g.9286T>C	MT-CO3	6771	11.79	1989	0.2	4904	5.69	Yes
P_11337a	g.9429G>A	MT-CO3	16,236	3.47	404	0	4975	3.50	Yes
P_5960a	g.9497T>C	MT-CO3	10,947	3.27	2603	0	4961	2.80	Yes
P_11336a	g.9594C>T	MT-CO3	10,659	5.65	623	0.16	4845	5.49	Yes
P_9567a	g.9645G>A	MT-CO3	11,647	6.36	700	0.14	5021	3.94	Yes
P_9847a	g.10177G>A	MT-ND3	22,790	3.05	18,685	0.02	3403	7.35	Yes
P_7426a	g.10463T>C	MT-TR	9817	99.98	1300	99.92	7	100.00	Yes
P_9757a	g.10747T>C	MT-ND4L	6998	7.82	2229	0.04	4963	10.50	Yes
P_11336a	g.10838A>G	MT-ND4	10,950	9.52	617	0	5006	5.81	Yes
P_5936a	g.11195G>A	MT-ND4	7126	8.73	3615	0.03	4923	7.39	Yes
P_9582a	g.11477G>A	MT-ND4	11,726	3.77	12,886	0.02	4963	5.00	Yes
P_7206a	g.11825G>A	MT-ND4	12,293	3.54	1094	0.09	4999	5.82	Yes
P_4266a	g.11984T>C	MT-ND4	14,006	13.17	8778	0.03	4951	9.41	Yes
P_9755a	g.12154C>T	MT-TH	8116	3.7	1603	0.06	263	8.75	Yes
P_7221a	g.12618G>A	MT-ND5	9056	6.1	2555	0.04	2853	8.24	Yes
P_8981a	g.12769G>A	MT-ND5	10,621	7.06	3108	0.03	4976	8.16	Yes
P_5930a	g.12771G>A	MT-ND5	10,265	11.83	3150	0	4972	9.61	Yes
P_6413a	g.12977T>C	MT-ND5	7340	34.1	2123	0.09	4942	2.47	Yes
P_4847a	g.13099G>A	MT-ND5	13,805	5.16	4972	0.02	4931	1.60	Yes
P_7409a	g.13156C>T	MT-ND5	8790	4.55	2599	0.04	4891	7.56	Yes
P_5946a	g.13178G>A	MT-ND5	5740	8.89	2846	0.07	3483	11.08	Yes
P_8979a	g.13198G>A	MT-ND5	11,789	8.59	2741	0	2877	7.92	Yes
P_11336a	g.13272C>A	MT-ND5	9660	5.48	579	0	4603	2.00	Yes
P_8979a	g.13496C>A	MT-ND5	10,985	5.5	2332	0.04	3916	5.80	Yes
P_11374a	g.13531G>A	MT-ND5	4731	5.14	1252	0	5007	3.87	Yes
P_11391a	g.13567A>G	MT-ND5	1322	23.83	856	0	5009	0.86	Yes
P_11372a	g.14112C>A	MT-ND5	8553	10.1	1251	0.08	4959	10.71	Yes
P_8611a	g.14197T>C	MT-ND6	10,577	5.01	4713	0.04	5005	5.97	Yes
P_7215a	g.14447T>C	MT-ND6	13,776	3.47	2242	0	2321	2.76	Yes
P_11374a	g.14760G>A	MT-CYB	5961	19.28	1421	0.07	921	0.00	No
P_4959a	g.14788T>C	MT-CYB	13,250	10.17	8208	0.12	4608	6.45	Yes
P_9570a	g.14888G>A	MT-CYB	15,466	3.84	517	0	3649	5.10	Yes
P_5954a	g.14939T>C	MT-CYB	8033	3.39	2633	0.04	2959	6.69	Yes
P_4982a	g.15012T>C	MT-CYB	22,666	8.39	4348	0.05	5016	7.93	Yes
P_5950a	g.15093G>A	MT-CYB	8082	5.26	1601	0	5037	7.72	Yes
P_9582a	g.15170G>A	MT-CYB	11,759	3.46	13,082	0.02	3899	9.69	Yes
P_11342a	g.15242G>A	MT-CYB	11,745	3.32	373	0	4339	6.04	Yes
P_9582a	g.15854T>C	MT-CYB	10,545	6.89	12,711	0.06	3895	7.01	Yes
P_4977a	g.15970T>C	MT-TP	19,783	99.96	5232	100	3	100.00	Yes
P_4847a	g.16033G>A	Control-Region	14,332	7.59	5556	0.02	2732	0.48	Yes
P_7433a	g.16147C>T	Control-Region	15,174	11	3874	0.03	2873	1.98	Yes
P_9539a	g.16293A>G	Control-Region	2683	9.58	470	0.21	3315	9.20	Yes

**Table A3:** Variants at only the mtRNA level.

Sample	Variant	Gene	Class	Depth RNA Tumor	VAF RNA Tumor	Comment
P_4982a	r.72u>c	Control-Region	Somatic	318	20.44	True variant (mutually exclusive 73G, 94T)
P_6406a	r.72u>c	Control-Region	Germline	1906	99.79	True variant
P_9589a	r.73a>g	Control-Region	Germline	592	99.83	True variant
P_5959a	r.73a>g	Control-Region	Germline	954	100.00	True variant
P_11394a	r.73a>g	Control-Region	Germline	923	100.00	True variant
P_11389a	r.146u>c	Control-Region	Germline	694	99.71	True variant
P_8978a	r.146u>c	Control-Region	Germline	371	99.73	True variant (phased with 185A and 204C)
P_8611a	r.146u>c	Control-Region	Germline	1599	99.75	True variant (phased with 195C)
P_8981a	r.146u>c	Control-Region	Germline	308	100.00	True variant
P_8609a	r.152u>c	Control-Region	Somatic	1448	69.96	True variant (phased with 195C)
P_10014a	r.152u>c	Control-Region	Germline	2272	95.38	True variant
P_4606a	r.152u>c	Control-Region	Germline	98	98.98	True variant
P_4266a	r.152u>c	Control-Region	Germline	956	99.37	True variant
P_8618a	r.152u>c	Control-Region	Germline	437	99.77	True variant
P_8979a	r.152u>c	Control-Region	Germline	371	100.00	True variant
P_4261a	r.182c>u	Control-Region	Somatic	1220	77.54	True variant
P_11383a	r.185g>a	Control-Region	Germline	301	99.67	True variant (phased with 150T and 228A)
P_5928a	r.185g>a	Control-Region	Germline	723	100.00	True variant (phased with 188G and 228A)
P_9592a	r.188a>g	Control-Region	Germline	814	99.63	True variant (phased with 185A and 228A)
P_5928a	r.188a>g	Control-Region	Germline	716	100.00	True variant (phased with 185A and 228A)
P_5956a	r.188a>g	Control-Region	Germline	1450	100.00	True variant (phased with 185A and 228A)
P_9571a	r.188a>g	Control-Region	Germline	37	100.00	True variant (phased with 185A, 222T and 228A)
P_4069a	r.189a>g	Control-Region	Germline	997	100.00	True variant
P_11372a	r.195u>c	Control-Region	Germline	715	99.72	True variant (phased with 152C and 263G)
P_8978a	r.228g>a	Control-Region	Somatic	573	82.72	True variant (phased with 185A, 204C, 263G)
P_11383a	r.228g>a	Control-Region	Germline	585	99.83	True variant (phased with 185A, 263G, 295T)
P_9597a	r.263a>g	Control-Region	Germline	2341	99.83	True variant (phased with 228A and 295T)
P_10010a	r.263a>g	Control-Region	Germline	1331	99.85	True variant
P_7238a	r.263a>g	Control-Region	Germline	1388	99.86	True variant (phased with 295T)
P_8611a	r.263a>g	Control-Region	Germline	1948	99.95	True variant (phased with 195C)
P_8830a	r.263a>g	Control-Region	Germline	892	100.00	True variant (phased with 207A and 234G)
P_7238a	r.295c>u	Control-Region	Germline	766	99.48	True variant (phased with 263G)
P_5956a	r.295c>u	Control-Region	Germline	1121	99.91	True variant (phased with 263G)
P_6732a	r.295c>u	Control-Region	Germline	545	100.00	True variant
P_9597a	r.295c>u	Control-Region	Germline	1272	100.00	True variant (phased with 228A and 263G)
P_7316a	r.456c>u	Control-Region	Germline	88	95.45	True variant
P_9758a	r.1604g>a	MT-TV	Somatic	159	23.27	True variant
P_11399a	r.1669g>a	MT-TV	Somatic	534	41.20	True variant
P_6730a	r.1973g>a	MT-RNR2	Somatic	1171	57.05	True variant
P_4977a	r.2166c>u	MT-RNR2	Somatic	1433	18.42	Potential artefact; at start of read ACCxATA context
P_10010a	r.2300g>a	MT-RNR2	Somatic	2066	13.84	True variant (in DNA! 2300G>A, 9106|5497|37.64%)
P_7431a	r.2416u>c	MT-RNR2	Somatic	855	84.80	True variant
P_6406a	r.3109u>c	MT-RNR2	Somatic	1407	38.38	True variant
P_4963a	r.3283g>a	MT-TL1	Somatic	908	29.52	True variant
P_4606a	r.3535u>c	MT-ND1	Somatic	2203	92.74	True variant
P_4080a	r.3705g>a	MT-ND1	Somatic	51	94.12	True variant
P_5942a	r.3796a>g	MT-ND1	Germline	2032	98.43	True variant
P_9754a	r.3913g>a	MT-ND1	Somatic	1685	12.11	True variant
P_7219a	r.4282g>a	MT-TI	Somatic	76	35.53	True variant
P_6733a	r.4360g>a	MT-TQ	Somatic	54	40.74	True variant
P_6728a	r.4408g>a	MT-TM	Somatic	199	17.09	True variant
P_6043a	r.4986a>g	MT-ND2	Somatic	2073	39.22	True variant
P_4847a	r.5479u>c	MT-ND2	Somatic	890	13.03	Potential artefact; at end of read TCCxACC context
P_9567a	r.6569c>u	MT-CO1	Somatic	1773	88.72	True variant
P_4970a	r.7045u>c	MT-CO1	Somatic	761	12.22	True variant
P_9847a	r.7146a>g	MT-CO1	Germline	757	98.15	True variant
P_9757a	r.7579u>c	MT-TD	Somatic	109	16.51	True variant
P_11383a	r.7698u>c	MT-CO2	Somatic	2277	12.60	True variant
P_9541a	r.7765a>g	MT-CO2	Somatic	797	17.69	True variant
P_4976a	r.7895u>c	MT-CO2	Somatic	2368	28.08	True variant
P_11819a	r.8149a>g	MT-CO2	Somatic	660	92.58	True variant
P_7216a	r.8286u>c	Non-Coding	Somatic	364	49.73	True variant
P_8978a	r.8408c>u	MT-ATP8	Germline	2239	99.60	True variant
P_4604a	r.9989u>c	MT-CO3	Germline	302	98.68	True variant
P_4833a	r.10306a>c	MT-ND3	Somatic	1004	35.26	True variant
P_4976a	r.11718g>a	MT-ND4	Somatic	1304	92.33	True variant
P_6722a	r.11899u>c	MT-ND4	Germline	691	95.37	True variant (phased with 11914A)
P_8978a	r.12763g>a	MT-ND5	Somatic	1602	89.20	True variant
P_9754a	r.12876c>u	MT-ND5	Germline	1478	97.29	True variant
P_6411a	r.13528a>g	MT-ND5	Somatic	1701	91.59	True variant
P_4983a	r.13552g>a	MT-ND5	Somatic	1569	91.65	True variant
P_9002a	r.14389c>u	MT-ND6	Somatic	1718	93.95	True variant
P_5930a	r.14721g>c	MT-TE	Somatic	302	27.81	True variant (phased with 14766T)
P_8978a	r.15495u>c	MT-CYB	Somatic	1812	94.09	True variant
P_11337a	r.15607a>g	MT-CYB	Germline	959	99.06	True variant
P_4982a	r.15904c>u	MT-TT	Somatic	344	34.01	True variant (overlapping 15927A)
P_4982a	r.15927g>a	MT-TT	Somatic	344	36.92	True variant (overlapping with 15904)
P_4847a	r.16092u>c	Control-Region	Germline	1838	99.78	True variant
P_11389a	r.16093u>c	Control-Region	Somatic	745	12.62	True variant
P_9582a	r.16093u>c	Control-Region	Somatic	753	12.88	True variant (phased with 16126C)
P_4072a	r.16093u>c	Control-Region	Germline	1041	27.76	True variant
P_9567a	r.16093u>c	Control-Region	Somatic	2459	92.19	True variant
P_4955a	r.16093u>c	Control-Region	Germline	1298	98.69	True variant
P_4970a	r.16104c>u	Control-Region	Germline	800	81.88	True variant
P_8979a	r.16184c>u	Control-Region	Germline	317	100.00	True variant
P_4606a	r.16186c>u	Control-Region	Germline	181	96.69	True variant
P_8830a	r.16209u>c	Control-Region	Germline	863	100.00	True variant (phased with 16171A, 16183C, 16188C, 16233T, 16258T)
P_8830a	r.16223c>u	Control-Region	Germline	1197	100.00	True variant (phased with 16171A, 16183C, 16188C, 1609C, 16258T)
P_9002a	r.16235a>g	Control-Region	Germline	1409	82.82	True variant (phased with 16183C, 16184A, 16189C, 16217C)
P_11338a	r.16235a>g	Control-Region	Somatic	2188	93.01	True variant (phased with 16293G and 16304C)
P_6730a	r.16267c>u	Control-Region	Somatic	2167	34.98	True variant
P_6732a	r.16278c>u	Control-Region	Somatic	2661	47.35	True variant
P_9575a	r.16290c>u	Control-Region	Somatic	1530	25.16	True variant (phased with 16265C, 16291T, 16335G)
P_11341a	r.16293a>g	Control-Region	Germline	1205	100.00	True variant (phased with 16331C, 16354T)
P_9582a	r.16294c>u	Control-Region	Germline	870	99.89	True variant (phased with 16304C)
P_9847a	r.16294c>u	Control-Region	Germline	731	100.00	True variant (phased with 16278T, 16293G, 16311C, 16360T)
P_9582a	r.16304u>c	Control-Region	Germline	663	99.85	True variant (phased with 16294T)
P_9596a	r.16311u>c	Control-Region	Germline	802	98.63	True variant
P_5956a	r.16311u>c	Control-Region	Germline	757	100.00	True variant
P_9761a	r.16336g>a	Control-Region	Germline	1571	100.00	True variant
P_9541a	r.16342u>c	Control-Region	Germline	806	99.75	True variant
P_11381a	r.16356u>c	Control-Region	Germline	1466	99.86	True variant
P_9568a	r.16362u>c	Control-Region	Germline	1232	99.84	True variant (phased with 16304C)
P_5946a	r.16362u>c	Control-Region	Germline	327	100.00	True variant
P_9599a	r.16362u>c	Control-Region	Germline	376	100.00	True variant (phased with 16325C)
P_11819a	r.16362u>c	Control-Region	Germline	373	100.00	True variant
P_10010a	r.16519u>c	Control-Region	Somatic	732	56.15	True variant
P_6730a	r.16540c>u	Control-Region	Somatic	1142	61.56	True variant (phased with 16519C)
