# Supplementary material for: Mitochondrial RNA Expression and Single Nucleotide Variants in Association with Clinical Parameters in Primary Breast Cancers
Source: Cancers (Basel). 2018 Dec 9;10(12):500. doi: 10.3390/cancers10120500 (PMC6318759; doi:10.3390/cancers10120500)
Supplement: Supplementary file 1 [file cancers-10-00500-s001.zip › cancers-396169-final-supplementary figures.docx]

**Supplementary Figures: Mitochondrial RNA Expression and Single Nucleotide Variants in Association with Clinical Parameters in Primary Breast Cancers**

Marjolein J. A. Weerts *, Marcel Smid, John A. Foekens, Stefan Sleijfer and John W. M. Martens

Department of Medical Oncology and Cancer Genomics Netherlands, Erasmus MC Cancer Institute,
Erasmus University Medical Center, 3015 CE Rotterdam, The Netherlands


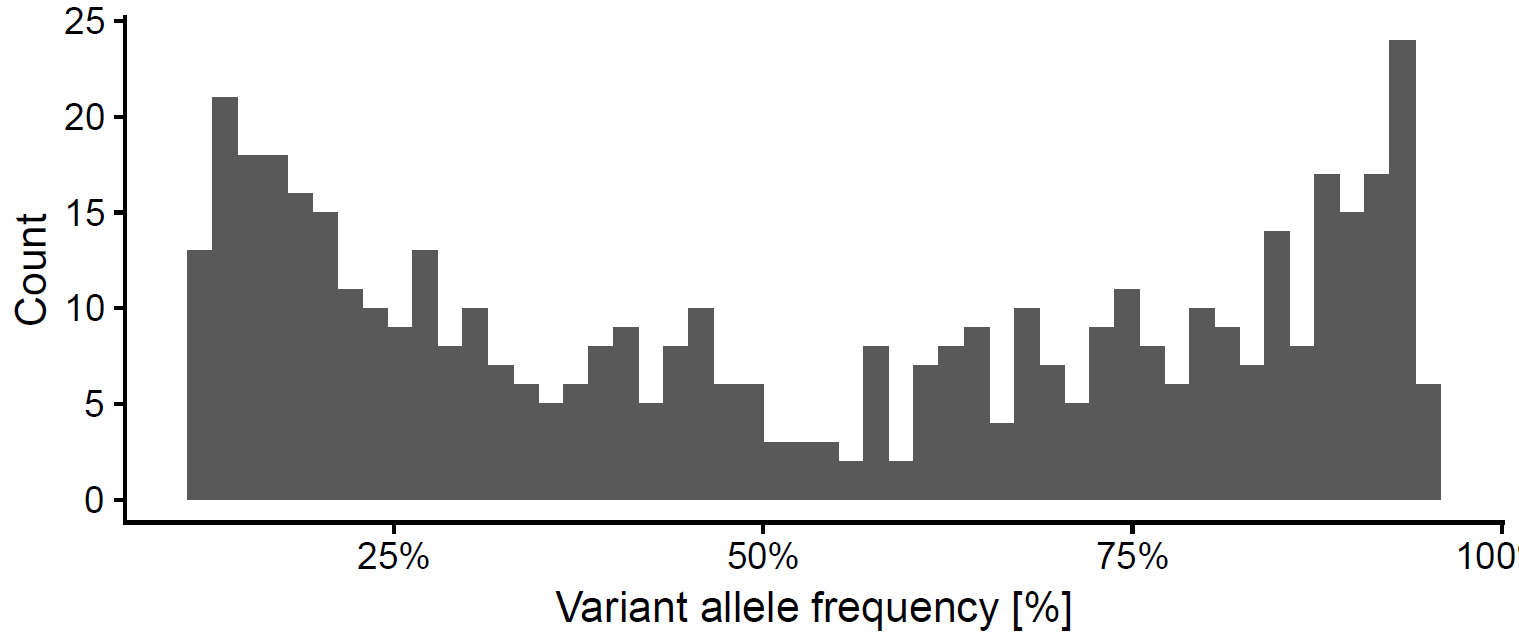


100%

**Figure S1.** Histogram of variant allele frequency (horizontal) of the somatic mitochondrial RNA variants of 344 primary breast tumor cases.

**
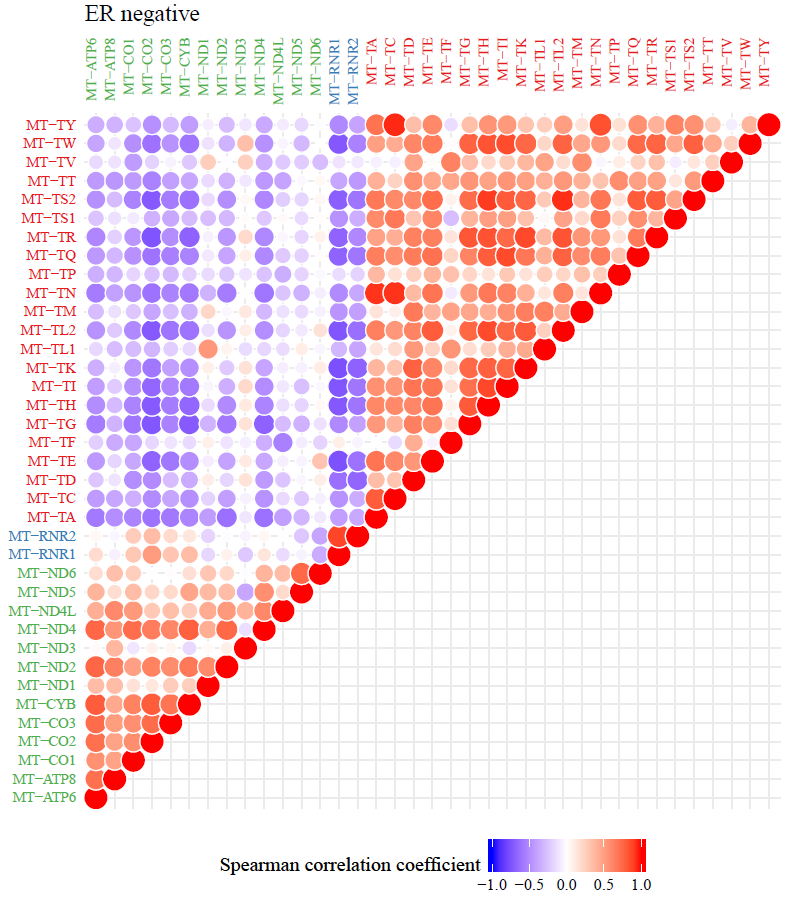
**

**Figure S2.** Correlation matrix of expression of all 37 mitochondrial-encoded genes of 81 ER-negative primary breast tumor cases. Correlation matrix depicting the Spearman correlation between all 37 mitochondrial-encoded genes (text of tRNA genes in red, rRNA genes in blue, mRNA genes in green). Color intensity and the size of the circle are proportional to the correlation coefficients.


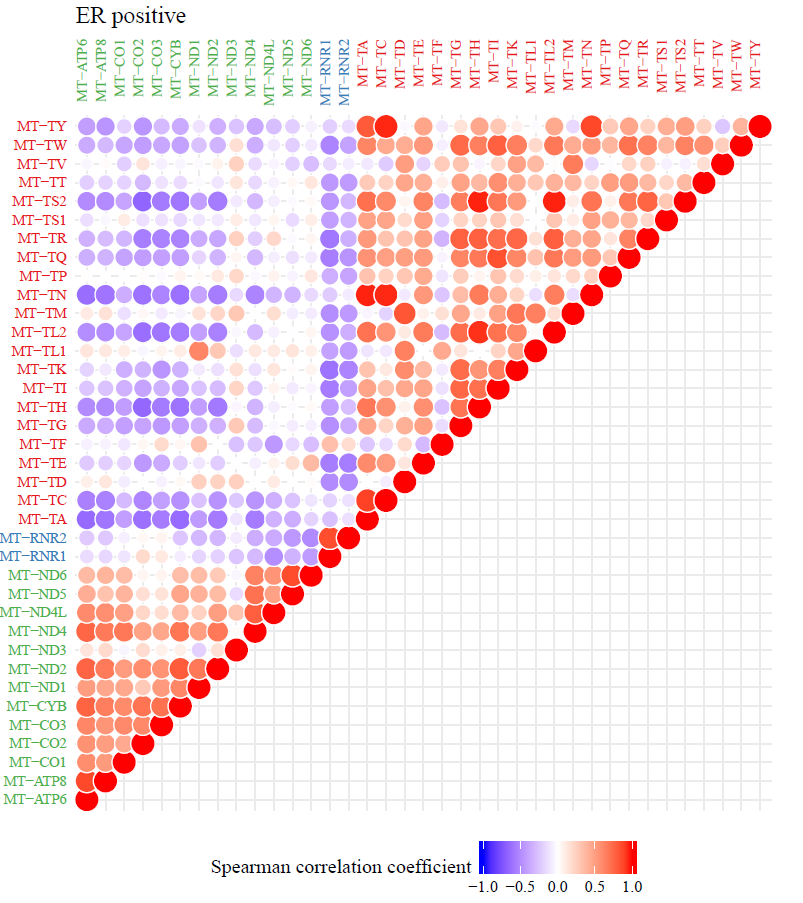


**Figure S3.** Correlation matrix of expression of all 37 mitochondrial-encoded genes of 210 ER-positive primary breast tumor cases. Correlation matrix depicting the Spearman correlation between all 37 mitochondrial-encoded genes (text of tRNA genes in red, rRNA genes in blue, mRNA genes in green). Color intensity and the size of the circle are proportional to the correlation coefficients.
